# 

**Published:** 1829-01-01

**Authors:** 


### CONTENTS

OF THE

### MEDICO-CHIRURGICAL REVIEW.

No. XIX. JANUARY 1, 1829.

### REVIEWS.

I.

Commentaries on the Causes Forms, Symptoms, and Treatment of Insanity. By G. M. BURROWS, M.D... .. .. .. .. .. .. .. .. .. .. .. .. .. .. 1

II.

HASTINGS—MALVERN—LEAMINGTON.

1. Dr. HARWOOD on the Curative Influence of the Southern Coast of England, especially that of Hastings .. .. .. .. .. .. .. .. .. .. .. .. .. 24
2. Mr. ADDISON on the Nature and Properties of the Malvern Waters, &c. .. 24
3. Dr. LOUDON on the Waters of Leamington Spa .. .. .. .. .. .. .. 24

III.

A Pathological Inquiry into the Nature of Hydrocephalus, grounded on Symptoms and Morbid Anatomy. By THOMAS MILLS, M.D. .. .. .. .. .. .. .. .. 29

IV.

Deafness, its Causes, Prevention and Cure. By J. STEVENSON, Esq. .. .. .. 45

V.

An Essay on Intermittent and Remittent Diseases, &c. By Dr. MACCULLOCH. Art. III.—NEURALGIA .. .. .. .. .. .. .. .. .. .. .. .. .. .. 49

VI.

Elements of Descriptive and Practical Anatomy. By JONES QUAIN, A.B. M.B. &c. 67

VII.

Cases and Observations, illustrating the Origin of Tubercles. By Professor ALISON 72

VIII.

Medical Essays, on Fever, Inflammation, Rheumatism, Diseases of the Heart, &c. By JOSEPH BROWN, M.D. .. .. .. .. .. .. .. .. .. .. .. .. 85

IX.

A Practical Treatise on Parturition. By SAMUEL ASHWELL .. .. .. .. .. .. 97

X.

Elements of the Theory and Practice of Medicine. By GEORGE GREGORY, M.D. 115

XI.

The Morbid Anatomy of the Bowels, Liver, and Stomach. By JOHN ARMSTRONG, M.D. [Fascic. Second.] .. .. .. .. .. .. .. .. .. .. .. .. .. 118

XII.

Observations on the Nature and Treatment of Cholera, and on the Pathology of the Mucous Membranes. By A. CHRISTIE, M.D. .. .. .. .. .. .. .. .. 123

XIII.

An Essay on the Treatment of Diseased Joints. By THOMAS BUCHANAN, C.M. .. 128

XIV.

On the Diagnosis of Dislocations and Fractures of the upper Extremity of the Humerus. By Baron DUPUYTREN .. .. .. .. .. .. .. .. .. .. .. .. 133

### PERISCOPE; OR CIRCUMSPECTIVE REVIEW.

Death of Dr. Gall .. 190
Lithontripteur greatly improved .. 191
Curious Action of Colchicum on the Urine .. 192
On Intemperance, as a Disease, and as susceptible of Cure. By Dr. Kain .. 193
Mr. Battley's Experiments on Aloes .. 195
Curious Cerebral or Nervous Affection, of a Periodical Character. By Dr. Strambio 195
Observations on Angina Pectoris. By Dr. Farre and others; with Comments .. 196
Influence of the Brain and Nervous System in the Production of Inflammation. By Dr. Malden .. 198
Iatraleptic Treatment of Dropsy .. 199
Enlargement of the Uterus cured by Iodine. By Dr. Thetford .. 200
Rapidly fatal Puerperal Peritonitis—Atmospheric Constitution of 1825-6-7-8. By Dr. Farre .. 201
Aneurism of the Middle Meningeal Artery—fatal operation .. 203
Death from Stricture .. 204
An Account of the Dandy, or Rheumatic Fever of the West Indies. By Dr. Stedman 205
Climate of Barbadoes—Phthisis .. 207
Scirrho-contracted Rectum—Valvular Obstruction—with Comments .. 208
Apoplexy caused (as was supposed) by Gymnastic Exercises, with Criticisms .. 211
Severe Affection of the Stomach and Duodenum, imitating Scirrhus of the Pylorus 211
Dr. Wilson on the Cure of Neuralgia .. 212
On Medical Examinations .. 241
Case of Lumbar Abscess in a Horse .. 243
Case of Spinal Neuralgia .. 244
Pulmonary Consumption—Quackeries of St. John Long .. 245
Toxicological Considerations on Local and General Blood-letting .. 248
On Medical Reporting .. 249
On the Treatment of Varicose Veins .. 250
Diagnostic Symptoms of Dislocation of the Head of the Femur .. 251
Curious Case of Periodical Vino-mania .. 253
Remarkable Case of Ischuria Renalis .. 254
Quacks and Quack Medicines .. 254
Amputation of Fungus Hæmatodes of the Breast .. 255
The Spine-Tinker .. 256

### THE HOSPITAL REPORTER, OR ECLECTIC CLINICAL REVIEW.

### Hotel Dieu.

Eclectic and Clinical Review of the Subject of Traumatic Gangrene, with Cases, Illustrations, and Criticisms .. 145
Dr. Andry on the Gangrena Senilis, or Dry Gangrene of Pott, with an Account of the successful Practice pursued by Baron Dupuytren .. 151
Case of Urinary Bladder mistaken for an Aneurism of the Aorta, at the Hôtel Dieu, 152
Memoir on Phlegmonous Tumours occupying the Right Iliac Fossa. By Baron Dupuytren .. 217

### Guy's Hospital.

Injection of the Cavity of a Psoas Abscess, with Criticisms .. 153
Compound Fracture of the Tibia and Fibula—Difficulty of Reduction—Amputation; with Criticisms .. 154

Mr. Amesbury's Practice in Fractures .. .. .. .. .. .. .. .. .. .. .. .. .. .. 235

LA CHARITE.

Mysterious Epidemic in Paris, bearing some resemblance to the Dandy Fever .. 155

HOSPITAL OF SURGERY.

Enormous Exostosis of the Sternum converted into Adipocere.. .. .. .. .. .. 157

HÔPITAL DE LA PITIE.

On the Treatment of Cancerous Affections of the Cervix Uteri; and especially on Amputation of that Part. By M. Lisfranc. With numerous Cases. .. .. 159

On the Treatment of Varicose Ulcers, with Cases and Commentaries .. .. .. 162

Cases of Paraplegia, which were supposed to be Epidemic in Paris .. .. .. 164

GLASGOW ROYAL INFIRMARY.

Dr. Anderson's Report on Fractures, with Cases and Observations.. .. .. .. 165

Hernia.. .. .. .. .. .. .. .. .. .. .. .. .. .. .. .. .. .. .. .. .. .. 166

Displacement of the Vertebræ .. .. .. .. .. .. .. .. .. .. .. .. .. .. 166

Ulcers.. .. .. .. .. .. .. .. .. .. .. .. .. .. .. .. .. .. .. .. .. .. 167

Hospital Gangrene .. .. .. .. .. .. .. .. .. .. .. .. .. .. .. .. .. .. 167

Urinary Abscess.. .. .. .. .. .. .. .. .. .. .. .. .. .. .. .. .. .. .. 168

Observations on Amputation .. .. .. .. .. .. .. .. .. .. .. .. .. .. .. 168

Erysipelas—Effects of Incisions .. .. .. .. .. .. .. .. .. .. .. .. .. .. 236

Burns and Scalds .. .. .. .. .. .. .. .. .. .. .. .. .. .. .. .. .. .. 237

Hydrocele .. .. .. .. .. .. .. .. .. .. .. .. .. .. .. .. .. .. .. .. .. 238

Fatal Results of Extirpation of the Breast .. .. .. .. .. .. .. .. .. .. .. 238

On Compound Fractures.. .. .. .. .. .. .. .. .. .. .. .. .. .. .. .. .. 262

Laryngitis—Bronchotomy .. .. .. .. .. .. .. .. .. .. .. .. .. .. .. .. 263

MONTPELLIER HOSPITAL.

M. Lallemand on Visceral Abscesses (Purulent Depôts) after Traumatic Lesions .. 169

SAINT GEORGE'S HOSPITAL.

Cancer of the Tongue—apparently fatal Effects of Arsenic exhibited for the Cure, 170

Case of Cut-throat—Symptoms of Cerebral Disease .. .. .. .. .. .. .. .. 172

Curious Enlargement of the Left Cheek—Anomalous Pain in the Head, &c... .. 172

SAINT THOMAS'S HOSPITAL.

On Encysted Tumours of the Scalp, with Cases and Remarks .. .. .. .. .. .. 173

Dislocation of the Thigh-bone into the Ischiatic Notch .. .. .. .. .. .. .. 238

Dislocation of the Radius forwards and upwards, with Fracture of the Ulna near the Olecranon .. .. .. .. .. .. .. .. .. .. .. .. .. .. .. .. .. .. 240

TOULON HOSPITAL.

General Induration of the Arterial System .. .. .. .. .. .. .. .. .. .. 174

ROYAL INFIRMARY OF EDINBURGH.

Dr. Ballingall on the Treatment of Fractures and Dislocations.. .. .. .. .. .. 175

A. Case of double Fracture of the Tibia .. .. .. .. .. .. .. .. .. 175

B. Ununited Fracture of the Humerus .. .. .. .. .. .. .. .. .. 176

C. Dislocation of the Elbow-joint.. .. .. .. .. .. .. .. .. .. .. 176

D. Dislocation of the Radius backwards .. .. .. .. .. .. .. .. .. 177

CHRONIC AFFECTIONS OF THE JOINTS .. .. .. .. .. .. .. .. .. .. .. .. .. 178

*Case* 1. Incipient Disease of the Hip-joint .. .. .. .. .. .. .. .. .. 178

2. Disease of Hip-joint, with Elongation .. .. .. .. .. .. .. .. 178

Cancerous Tumours .. .. .. .. .. .. .. .. .. .. .. .. .. .. .. .. .. .. 226

Polypus Uteri .. .. .. .. .. .. .. .. .. .. .. .. .. .. .. .. .. .. .. 227

Popliteal Aneurism—Operation .. .. .. .. .. .. .. .. .. .. .. .. .. .. 228

LONDON FEVER HOSPITAL.

Dr. Tweedie on a peculiar Swelling of the Lower Extremity, post Febrem .. .. 180

CHELTENHAM CASUALTY HOSPITAL.

On the Removal of Loose Cartilages from the Knee-joint. By Charles Averill, Esq. 181

BELFAST HOSPITAL.

Gastric and diaphragmatic Perforations; with Medico-legal Remarks .. .. .. 221

ST. BARTHOLOMEW'S HOSPITAL.

Ulcerations of the Inguinal Glands and Penis, relieved by the Chlorate of Lime .. 222

CORK STREET FEVER HOSPITAL.

Remarkable Instance of Extra-uterine Fœtation .. .. .. .. .. .. .. 223

On Fever. By Dr. O'Reardon .. .. .. .. .. .. .. .. .. .. .. .. 259

CLINICAL INSTITUTION OF PARMA.

Ascites cured by Pressure .. .. .. .. .. .. .. .. .. .. .. .. .. 224

WESTMINSTER OPHTHALMIC HOSPITAL.

Report of Mr. Guthrie's Practice in Diseases of the Eye .. .. .. .. .. .. 224

WORCESTER INFIRMARY.

Tumour growing from the Uterus, &c. .. .. .. .. .. .. .. .. .. .. .. 229

WESTMINSTER HOSPITAL.

I. Lithotomy .. .. .. .. .. .. .. .. .. .. .. .. .. .. .. 231

II. Hydrocele, of different kinds .. .. .. .. .. .. .. .. .. .. .. 231

III. Amputations .. .. .. .. .. .. .. .. .. .. .. .. .. .. 233

MIDDLESEX HOSPITAL.

Curious Practice in a Case of Strangulated Hernia .. .. .. .. .. .. .. 234

MAISON ROYALE DE SANTE.

Observations on one of the most frequent Causes of Abortion. By Madame Boivin, M.D. .. .. .. .. .. .. .. .. .. .. .. .. .. .. .. 257

HOPITAL DU MANS.

Pathology of Traumatic Tetanus .. .. .. .. .. .. .. .. .. .. .. .. 261

SANTA MARIA HOSPITAL, FLORENCE.

Successful Case of Cæsarean Section .. .. .. .. .. .. .. .. .. .. .. 264

FALSE REPORTS.

Grossly erroneous Report of a Case in St. George's Hospital .. .. .. . .. 182

MEDICAL JURISPRUDENCE.

THE NEW LONDON UNIVERSITY.

Sketches of the Introductory Lectures of Bell, Conolly, Davis, Pattison, Thomson, Watson, and Turner; with general Remarks on the Objects, Tendency, and probable Consequences of this Institution.. .. .. .. .. .. .. .. .. 184

New Curriculum of the Apothecaries' Company, with Explanations and Comments 189

Insurance on Lives—Medical Certificates—Medical Evidence—Remarks on a recent Trial .. .. .. .. .. .. .. .. .. .. .. .. .. .. .. .. 213

Amputation and Extirpation of the Uterus; with Remarks on this Operation, and its Results .. .. .. .. .. .. .. .. .. .. .. .. .. .. .. .. 215

The AGE OF LIBEL—COOPER *v.* WAKLEY; with a Critical Examination of the Evidence, Arguments, Verdict, and Moral Consequences of this Trial

Investigation of the Causes of the Age of Libel .. .. .. .. .. .. .. .. 266

Full Report of the Trial .. .. .. .. .. .. .. .. .. .. .. .. .. .. 268

Critical Examination of the Evidence, Arguments, Verdict, &c .. .. .. .. 297

Bibliographical Record .. .. .. .. .. .. .. .. .. .. .. .. .. .. 306

Notice of Dr. Burrows' "Reclamation" .. .. .. .. .. .. .. .. .. .. 308

*N.B. The Periscopic and Clinical Articles are arranged separately.*

